# Effect of LED Lighting Regime on Quality of Pak Choi (*Brassica rapa* subsp. *chinensis*) During Cold Storage

**DOI:** 10.3390/foods15101669

**Published:** 2026-05-11

**Authors:** Boyu Mao, Mengdi Niu, Hao Liu, Jun Wang

**Affiliations:** College of Food Science & Nutritional Engineering, China Agricultural University, 17 Qinghua East Road, Beijing 100083, China

**Keywords:** pak choi, LED regime, refrigeration, nutritional quality, transcriptomic analysis

## Abstract

Light combined with refrigeration is an effective strategy for preserving postharvest fruit and vegetable quality. This study explored the effects of various red–blue (R/B) light-emitting diode (LED) light/dark (L/D) cycles on pak choi during refrigeration. Pak choi exposed to a 4/20 h L/D cycle exhibited greater freshness, along with the highest levels of total phenolic compounds, chlorophyll, and ascorbic acid, particularly after 6 days of refrigeration. Transcriptomic analysis showed that this lighting regime upregulated genes associated with phenylpropanoid and flavonoid biosynthesis (PAL, C4H, ANS, CHS, FLS) and ascorbate metabolism (VTC2_5, VTC4, APX), boosting phenolic and ascorbic acid content. It also reduced chlorophyll degradation by increasing the expression of genes in the porphyrin metabolism pathway (CRD1, POR, DVR). These findings highlight the benefits of R/B light with a 4/20 h L/D cycle for enhancing pak choi quality during refrigeration storage. This approach provides an efficient approach for vegetable preservation.

## 1. Introduction

Pak choi (*Brassica rapa* subsp. *chinensis* “Shanghai Green”), widely cultivated in East Asia [1], is a popular leafy vegetable valued for its low calorie content, high dietary fiber, and abundant minerals and vitamins [2]. However, pak choi is highly perishable after harvest due to its high moisture content and rapid respiration rate at room temperature [3]. Consequently, low-temperature storage, particularly refrigeration, is commonly employed to preserve postharvest quality and extend the shelf life of pak choi [4], owing to its convenience and low cost [5]. However, even under refrigeration, many fruits and vegetables continue to respire, resulting in quality deterioration and nutrient loss [6]. For example, during the cold storage of broccoli, the content of carotenoids, glucosinolates and other nutrients decreases remarkably [7]. Harvested plant leaves remain metabolically active and can undergo light-driven processes such as photosynthesis [8], which help delay chlorophyll degradation, promote sugar accumulation, and replenish respiratory substrates [9]. Against this background, light irradiation has emerged as a promising postharvest treatment for actively regulating physiological processes in fruits and vegetables, thereby enhancing the synthesis of certain nutrients. Light-emitting diodes (LEDs) are especially attractive due to their high energy conversion efficiency, compact size, and long service life [10]. Studies have demonstrated that red light treatment of tea plants can maintain high levels of chlorophyll and carotenoid content in tea leaves [11], and that short-term blue light treatment increases anthocyanin and flavonoid contents in Chinese cabbage during the shelf life [12]. Similarly, red and blue light LED treatment on postharvest tomato consistently improved the accumulation of ascorbic acid and antioxidant activity [13].

To date, most research on the application of LED light for postharvest storage has focused on the effects of various wavelengths and light intensities [14]. However, the impact of different light/dark (L/D) cycles, specifically [8] light intervals under a constant daily light integral (DLI), on the quality of refrigerated fruits and vegetables remains underexplored [15].

This study addresses this gap by investigating the effects of LED light with various light regimes under the same DLI on key physiological and nutritional indicators in pak choi during refrigeration. Parameters, including appearance, total phenolic content (TPC), ascorbic acid content (AAC), and total chlorophyll content (TCC), were assessed, and RNA sequencing (RNA-Seq) was performed to elucidate changes in gene expression in metabolic pathways governing these attributes. The findings provide mechanistic insight into how lighting regimes regulate physiological processes in pak choi and offer valuable guidance on the application of combined LED light and refrigeration for postharvest preservation of fruits and vegetables in household refrigerators.

## 2. Materials and Methods

### 2.1. Materials and Reagents

Fresh pak choi was planted in the Daxing district in Beijing (N: 39°43′, E: 116°20′), purchased from a local supermarket in Beijing, China, and transported to the laboratory within 2 h of purchase. The samples were carefully selected to ensure uniformity in size, color, and maturity, with an average weight of 25 ± 10 g per piece, and were free from visible mechanical damage, decay, or symptoms. Prior to treatment, all samples were gently cleaned to remove surface impurities and randomly divided into different experimental groups.

HPLC-grade ethanol (purity ≥ 99%), gallic acid (purity ≥ 99%), sodium carbonate (Na_2_CO_3_, purity ≥ 99%), ascorbic acid (purity ≥ 99%), and oxalic acid (purity ≥ 99%) reagents were purchased from J&K Scientific (Beijing, China). Folin phenol reagent (2N) and 2,6-dichlorophenolindophenol sodium salt (DCPIP, ≥97%) were purchased from Solarbio Science & Technology Co., Ltd. (Beijing, China). Analytical-grade hydrochloric acid (HCl) was purchased from Sinopharm Group Chemical Reagent Co., Ltd. (Shanghai, China). Ultrapure water was obtained from a Millipore Synergy UV system (Millipore, Bedford, MA, USA).

### 2.2. Sample Storage and LED Protocol

A total of 6 kg of pak choi was evenly divided into four groups and placed flatly in the refrigerator (Midea Group Co., Ltd., Foshan, China) at 4 ± 1 °C and 80–95% relative humidity for up to 8 days. All treatment groups, with the exception of the control group, were subjected to illumination by a combination of red (660 nm) and blue (450 nm) LED panels with a fixed position above the samples (10 cm). The total power of the LED panels was 600 mW, with a blue-to-red light ratio of 1:1. The light intensity irradiated onto the surface of the sample was 280–530 lux, and the DLI was established at the same level for all treatments (4 h day^−1^).

The selected parameters, including light source power, DLI value, and illumination intensity, employed in this study were determined based on the design specifications of the refrigerator and the service life of the LED panels. In addition, different light/dark (L/D) cycles were set for each treatment group and managed by specialized controllers (Xincheng Electronics Co., Ltd., Ningbo, China). Group A served as the control and was maintained in continuous darkness (without lighting) during the 8-day refrigeration process. Groups B and C were exposed to alternating light and dark modes, with a 10 min lighting and 50 min darkness cycle for Group B (10/50 min) and a 30 min lighting and 150 min darkness cycle for Group C (30/150 min), which were continuously repeated every 24 h per day to maintain the same total DLI. In contrast, Group D was subjected to a continuous 4 h light period followed by 20 h of darkness each day (4/20 h), which was repeated throughout the 8-day refrigeration period. Sampling occurred at 0, 2, 4, 6, and 8 days after treatment. At every time point, 6 pak choi were randomly selected from each group and divided into three independent biological replicates. Then the pak choi samples were immediately frozen in liquid nitrogen, homogenized with a high-speed tissue homogenizer, and stored at –20 °C for subsequent physiological analyses [16].

### 2.3. Determination of Pak Choi Appearance Change and Color Coefficient

Samples were removed from refrigeration and left at room temperature for 30 min before appearance testing. The appearance changes of pak choi samples were recorded by a camera in a small studio.

Color parameters, including L* (lightness), a* (green–red), and b* (yellow–blue), were measured using a Konica Minolta CR 400 Chroma Meter (Konica Minolta Sensing, Inc., Osaka, Japan) with a D65 light source, a 2° observer angle, and a circular measurement area [16].

### 2.4. Analysis of Physicochemical Properties of Pak Choi Under Different Lighting Regimes

#### 2.4.1. Moisture Content

Moisture content was measured using a moisture content analyzer (Sartorius AG, Göttingen, Germany). Samples were dehydrated at 105 °C until a constant weight was achieved. Moisture content was calculated as the percentage weight loss during drying.

#### 2.4.2. Total Phenolic Content

TPC was determined using the Folin phenol method of Vaezi [17], with a minor modification. Three grams of the sample powder was extracted with 25 mL of 70% acidic ethanol solution (pH 2.7, adjusted with HCl) using an ultrasonic instrument (Kunshan Ultrasonic Instruments Co., Ltd., Kunshan, China) at 30 °C for 30 min. The extraction was performed twice. Both extraction solutions, collected by centrifugation at 3500 r/min for 10 min using a centrifuge (Changsha Xiangyi Centrifuge Instrument Co., Ltd., Changsha, China), were combined in a 50 mL volumetric flask.

An aliquot of 0.1 mL of the combined extract was transferred to a 10 mL centrifuge tube, followed by the addition of 1 mL Folin phenol reagent and 3 mL of 15% (*w*/*w*) Na_2_CO_3_. The mixed solution was diluted to 10 mL with ultrapure water, shaken thoroughly, and incubated for 60 min at room temperature in the dark. Absorbance was measured at 765 nm using a spectrophotometer (Beijing Purkinje General Instrument Co., Ltd., Beijing, China). TPC was reported as milligrams of gallic acid equivalents per gram of fresh pak choi [18].

#### 2.4.3. Ascorbic Acid Content

AAC was determined using a modified DCPIP titration method [19]. Briefly, 1 mL of 1.0 mg/mL ascorbic acid standard solution was mixed with 10 mL of 20 g/L oxalic acid solution in an Erlenmeyer flask. The mixture was titrated with 0.2 mg/mL DCPIP solution until a persistent pink color appeared (approximately 15 s), and the volume consumed was recorded to calculate the titration factor (Equation (1)):

(1) $$T=\frac{c\;\times \;V}{V_{1}\;-{\;V}_{0}}$$

where *T* is the titer of DCPIP solution (mg/mL), *c* is the concentration of the ascorbic acid standard solution, *V*_1_ is the volume of DCPIP solution consumed for titrating ascorbic acid standard solution (mL), and *V*_0_ is the volume of the DCPIP solution consumed in a blank test (mL).

For sample analysis, 5.0 g of pak choi powder was extracted with 20 g/L oxalic acid solution using ultrasonication for 10 min. The extract was centrifuged at 4000 r/min for 5 min; this operation was repeated twice, and the supernatants were combined in a 25 mL volumetric flask. An aliquot of 10 mL supernatant was transferred to a flask and titrated with DCPIP solution as described above. The volume of titrant required to reach the endpoint was recorded, and AAC was calculated according to Equation (2):

(2) $$\begin{array}{c} AAC\;(\mathrm{mg}/100\;g)=\frac{\left(V\;-\;V_{0}\right)\;\times \;T\;\times \;A\;\times \;100}{m} \end{array}$$

where *V* is the volume of the DCPIP solution consumed in the titration of the test solution (mL), *V*_0_ is the volume of the DCPIP solution consumed in a blank test (mL), *T* is the titer of DCPIP solution (mg/mL), *A* represents the dilution times of the test solution, and *m* is the mass of test sample (g).

#### 2.4.4. Chlorophyll Content

Chlorophyll content was determined following a modified version of the method described by Gu [20]. Briefly, 1 g of sample powder was extracted with 50 mL of 95% ethanol solution for 4 h or until the tissue turned white. Then, the mixture was shaken three times and centrifuged at 10,000 r/min for 10 min at 4 °C to obtain the supernatant. The absorbance of the supernatant was measured at 649 and 665 nm using an ultraviolet–visible spectrophotometer [21], with 95% ethanol serving as the blank. TCC was calculated according to the following equations:

(3) $${Chlorophyll}_{a}=13.95A_{665}-6.88A_{649}$$

(4) $${Chlorophyll}_{b}=24.96A_{649}-7.32A_{665}$$

(5) $$TCC\;(mg/g)=\frac{\left(C_{a}+C_{b}\right)\times V\times N}{W}$$

where *A*_649_ and *A*_665_ are the absorbances of supernatant at the wavelengths of 649 and 665 nm, *V* is the volume of extraction solution (mL), *N* represents the dilution factor of the test solution, and *W* is the fresh weight of the pak choi samples (g).

### 2.5. Total RNA Extraction, Sequencing, and Transcriptomic Analysis

Total RNAs were extracted from pak choi samples using the MJZol Total RNA Extraction Kit (Major Bio-pharm Technology Co., Ltd., Shanghai, China) following the manufacturer’s protocol. Purity and concentration of the RNAs were assessed with a NanoDrop 2000 (Thermo Fisher Scientific, Waltham, MA, USA), and the integrity of all total RNA samples was verified by agarose gel electrophoresis. RNA samples from the control group (CG) and optimal light treatment group (EG) at 0, 2, 4, 6, and 8 days of refrigerated storage were used for cDNA synthesis and transcriptome sequencing, with three biological replicates per condition. cDNA libraries were constructed and sequenced on an Illumina NovaSeq X Plus platform (Majorbio Co., Ltd., Shanghai, China). After quality control, low-quality reads were discarded [22], and clean data samples were aligned to the pak choi reference genome (*B. rapa* subsp. *chinensis*) http://brassicadb.cn/#/Download/ (accessed on 5 January 2026). Sequence numbers and transcript lengths were normalized and quantified using String Tie software (Version 2.2.1). The expression levels of transcripts were assessed by counting the number of fragments per kilobase per ten million fragment mappings (FPKM). Differentially expressed genes (DEGs) were identified using DESeq2 (|log_2_FC| > 1, FDR < 0.05), and then functionally annotated.

### 2.6. Statistical Analysis

All experiments were performed in triplicate, and data are presented as means ± standard deviation (SD). Statistical significance was evaluated by one-way analysis of variance (ANOVA) using IBM SPSS Statistics 27 (SPSS Inc., Chicago, IL, USA), with significant differences indicated as *, *p* < 0.05; **, *p* < 0.01; and ***, *p* < 0.001. Histograms were generated using Origin Pro 2020 (Origin Lab Corp., Northampton, MA, USA). Transcriptomic results were analyzed using the Majorbio Cloud Platform https://cloud.majorbio.com/ (accessed on 10 December 2025).

## 3. Results and Discussion

### 3.1. Appearance

**Figure 1:**
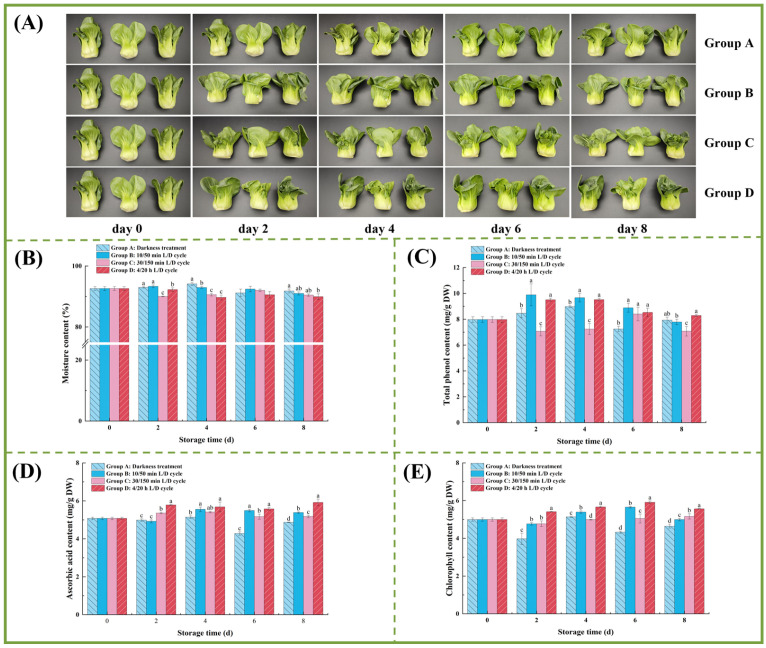
The appearance and key quality changes of pak choi under different light regimes. (**A**) The appearance changes of pak choi. (**B**) Moisture content. (**C**) Total phenol content. (**D**) Ascorbic acid content. (**E**) Chlorophyll content. Different letters indicate significant differences (*p* < 0.05) among groups on each refrigeration day.

Appearance quality is a primary determinant of consumer purchase decisions and an important indicator of freshness in vegetables [23]. As illustrated in Figure 1A, pak choi appearance changed over the 8 days of refrigerated storage. Group A exhibited pronounced yellowing and wrinkling of leaf edges, indicative of quality deterioration. In contrast, samples in Groups B, C, and D displayed minimal visual changes, with Group D (LED light exposure with a 4/20 h L/D cycle) maintaining the most vibrant green coloration among the groups.

Leaf color differences were quantitatively assessed using L*, a*, and b* values (Table 1). The L* value, representing brightness, remained unchanged in Groups A, B, and C, but progressively increased in Group D, reaching a maximum on day 8 of refrigerated storage, indicating enhanced brightness. Group D also exhibited consistently lower a* values, corresponding to a more intense green color, compared to the other groups at the same storage time. There were no significant differences in b* values among the treated groups. These quantitative results are consistent with the visual assessments in Table 1.

### 3.2. Changes in the Physicochemical Properties of Pak Choi

#### 3.2.1. Moisture Content Analysis

Moisture loss during low-temperature storage can adversely affect the taste and appearance of vegetables, which are critical indicators of vegetable quality [24]. As shown in Figure 1B, the moisture content of pak choi exhibited a slight but non-significant decline from day 0 to day 8, remaining at approximately 90% throughout the storage period. These findings indicate that the different lighting regimes did not substantially impact moisture retention during chilled storage.

#### 3.2.2. Total Phenolic Analysis

Phenolic compounds serve as natural antioxidants in plants, scavenging free radicals and bolstering defense against pathogens [25]. Most vegetables produce large amounts of reactive oxygen species (ROS) during postharvest refrigeration, leading to cell membrane oxidation and subsequent wilting; phenolic compounds help mitigate this oxidative stress [14].

TPC is closely linked to physiological properties, such as texture, enzyme activity, and respiration, and can be influenced by storage lighting conditions [26]. As depicted in Figure 1C, Groups B and D showed higher TPC than other groups from day 2 to day 4, although differences between these groups were not significant. By day 6, treated groups had significantly higher TPC than the control group, and by day 8, Group D maintained the highest TPC at 8.29 mg/g. While low-temperature storage generally delays the degradation of nutrients, such as TPC, in vegetables, in this study, exposure to mixed red and blue light with a 4/20 h L/D cycle (Group D) notably promoted phenolic compound accumulation (first 6 days) and slowed polyphenol loss (day 8) in pak choi during chilled storage.

In response to oxidative stress, plants activate polyphenol biosynthetic genes, temporarily increasing polyphenol content. While refrigeration is a common preservation method, its effect is only temporary. Although TPC generally declines with prolonged storage and senescence, our results suggest that specific lighting treatments, particularly Group D, can attenuate this decline. This may arise from the ability of specific lighting combinations and regimes to further promote the expression levels of genes associated with polyphenol synthesis, as demonstrated in Section 3.3.

#### 3.2.3. Ascorbic Acid Analysis

Ascorbic acid is a vital water-soluble vitamin for both plant physiology and human nutrition [27]. Figure 1D depicts the impact of different lighting regimes on the changes in AAC in pak choi during refrigerated storage. In Group D, AAC progressively increased, peaking at 5.92 mg/100 g on day 8, representing a 1.17-fold increase from baseline. Groups B and C displayed fluctuating ascorbic acid levels, but both maintained higher concentrations than the control group (*p* < 0.05) from day 6 to day 8.

These findings indicate that combined red and blue LED light can enhance ascorbic acid retention in pak choi during chilled storage. This observation is consistent with Song’s research [28]. Notably, our results further demonstrate that exposure to LED light with a 4/20 h L/D cycle is effective in promoting and sustaining elevated ascorbic acid levels under equivalent DLI conditions, with these findings also highlighting the importance of lighting regime selection for nutrient preservation in pak choi during chilled storage.

#### 3.2.4. Chlorophyll Contents Analysis

Like other leafy vegetables, pak choi exhibits a limited shelf life and is prone to yellowing due to chlorophyll degradation during transportation and storage, which affects both sensory quality and commercial value [29]. As shown in Figure 1E, the initial TCC in pak choi was 4.99 mg/g. Under control conditions (Group A), TCC generally declined, reaching its lowest point (86% of baseline) by day 6. Compared with Group A, the groups exposed to LED light showed a significant difference. Groups B and D exhibited a trend of increased TCC, followed by a decline. The content of chlorophyll in Group D was higher than in the other groups and was maintained at a high level of 5.56 mg/g on day 8. These results are consistent with previous studies showing that red and blue light irradiation can delay chlorophyll degradation in green vegetables such as butterhead lettuce, sweet peppers, and iceberg lettuce [30]. Our findings demonstrate that LED light with a 4/20 h L/D cycle (Group D) retards chlorophyll degradation in pak choi during refrigerated storage, which is similar to Pintos’s finding [31].

### 3.3. The Influence of LED Protocol on the Expression of DEGs in Different Nutrient Metabolic Pathways During Refrigeration of Pak Choi

The result of Lorena’s research was similar to ours. Compared to red or blue lighting treatment, the application of a combination of red and blue light irradiation significantly enhances the phytochemical accumulation in tomatoes during refrigeration [32]. Meanwhile, our research also demonstrated that adopting appropriate LED light regimes can significantly enhance the nutritional quality of pak choi, including TPC, AAC, and TCC. Among the treatments, LED light with a 4/20 h L/D cycle (Group D) produced a relatively obvious improvement in both appearance and nutritional quality. Light plays an important role in regulating postharvest plant metabolism and affecting plant chemical composition [33].

To elucidate the metabolic processes underlying these quality improvements, transcriptomic analyses were performed on samples from the control group (A: continuous darkness) and the optimal lighting group (D), focusing on DEGs.

#### 3.3.1. RNA-Seq Data and Quality Control

**Figure 2:**
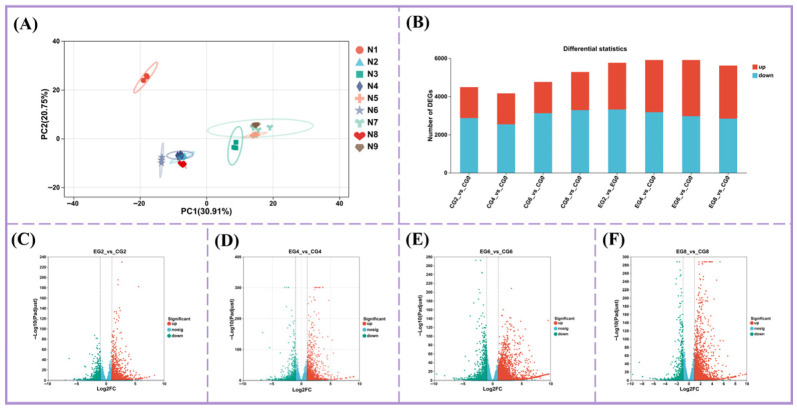
Identification and analysis of differentially expressed genes (DEGs) in refrigerated pak choi under the lighting mode of Group D. (**A**) Principal component analysis (PCA) of samples. (**B**) Statistical map of DEGs. (**C**) Volcano plots of DEGs for EG2 vs. CG2. (**D**) Volcano plots of DEGs for EG4 vs. CG4. (**E**) Volcano plots of DEGs for EG6 vs. CG6. (**F**) Volcano plots of DEGs for EG8 vs. CG8.

To further elucidate the molecular effects of Group D lighting, cDNA libraries were constructed from samples of Groups A and D at multiple storage time points and sequenced. The total clean data across 27 samples was 184.09 Gb, and the GC contents ranged from 46.56% to 47.01%. Quality metrics were high, with Q20 scores ranging from 98.09% to 99.06%, and Q30 scores ranging from 96.69% to 96.89%. Principal component analysis (PCA) revealed that PC1 accounted for 30.91% of the variance, clearly separating samples by treatment condition, while PC2 explained an additional 20.75% (Figure 2A). These results confirm the high quality and reliability of the transcriptomics data, supporting subsequent analyses.

Differential gene expression analysis revealed a total of 11,941 DEGs across 12 comparison groups: CG2 vs. CG0, CG4 vs. CG0, CG6 vs. CG0, CG8 vs. CG0, EG2 vs. CG0, EG4 vs. CG0, EG6 vs. CG0, EG8 vs. CG0, EG2 vs. CG2, EG4 vs. CG4, EG6 vs. CG6, and EG8 vs. CG8. In the control group (Group A), more DEGs were downregulated than upregulated as refrigeration time increased (CG2 vs. CG0, CG4 vs. CG0, CG6 vs. CG0, and CG8 vs. CG0). In contrast, Group D (4/20 h L/D cycle) showed a substantial increase in upregulated DEGs, particularly when comparing samples at the same storage time (EG2 vs. CG0, EG4 vs. CG0, EG6 vs. CG0, and EG8 vs. CG0). For example, in the EG6 versus CG6 comparison, 2802 genes were upregulated and 1224 downregulated on day 6 of refrigeration. These results indicate that the Group D lighting regime actively stimulates gene expression changes associated with physiological and metabolic processes during chilled storage.

#### 3.3.2. Analysis and Functional Annotation of DEGs

**Figure 3:**
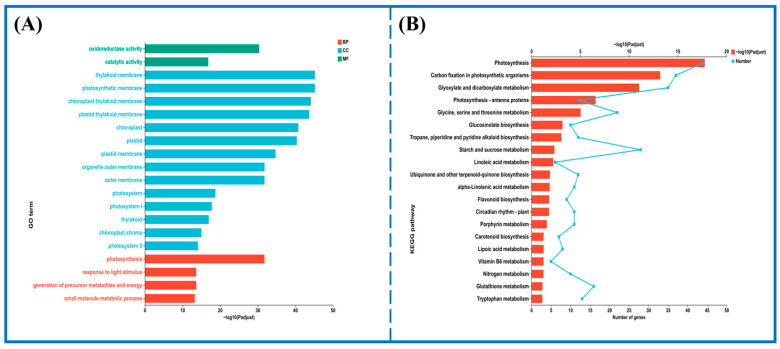
Gene Ontology (GO) and Kyoto Encyclopedia of Genes and Genomes (KEGG) analyses of DEGs between CG and EG in pak choi. (**A**) GO enrichment of DEGs. (**B**) KEGG pathway classification of DEGs.

To further elucidate the metabolic pathways responding to Group D lighting during refrigeration, DEGs from key comparison groups (CG2 vs. EG2, CG4 vs. EG4, CG6 vs. EG6, and CE8 vs. EG8) were subjected to GO and KEGG enrichment analyses. GO annotation analysis revealed that the most significantly affected terms in the biological process (BP) category were “photosynthesis,” “small molecule metabolic process,” and “organic acid metabolic process.” In the molecular function (MF) category, the dominant subcategories were “oxidoreductase activity,” “catalytic activity,” and “tetrapyrrole binding.” In the cellular components (CC) category, the dominant subcategories included “chloroplast,” “plastid,” and “thylakoid membrane” (Figure 3A).

KEGG enrichment analysis (Figure 3B) showed significant enrichment of DEGs in pathways associated with glucosinolate biosynthesis, carotenoid biosynthesis, phenylpropanoid biosynthesis, and flavonoid biosynthesis. Additionally, given their central role in postharvest quality, the ascorbate and aldarate metabolism pathway and the porphyrin metabolism pathway were also specifically examined for differential gene expression patterns.

#### 3.3.3. Key Gene Expression Related to Phenylpropanoid and Flavonoid Synthesis

**Figure 4:**
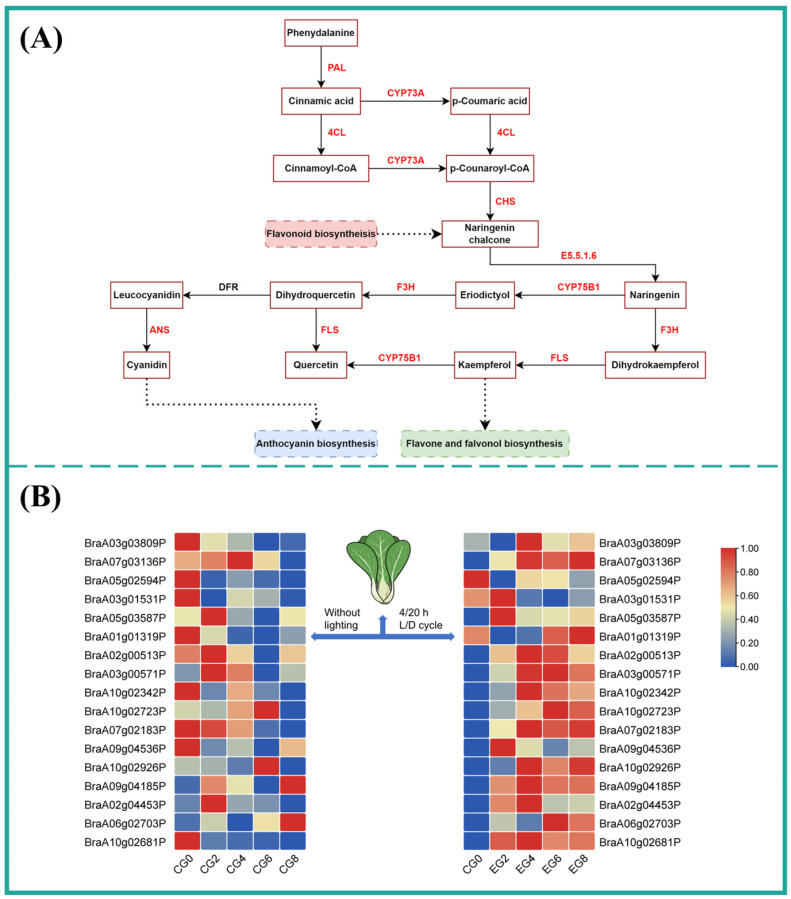
Effects of lighting regime of Group D on gene expression in phenylpropanoid and flavonoid biosynthesis in pak choi. (**A**) DEGs associated with phenylpropanoid and flavonoid biosynthesis pathways. (**B**) Heat map of DEG changes in phenylpropanoid and flavonoid biosynthesis during refrigeration. Red/blue fonts indicate upregulated/downregulated DEGs in the comparison of CK8 vs. EG8; the same applies below.

Changes in TPC in pak choi were closely related to the phenylpropanoid and flavonoid biosynthesis pathways. Transcriptome analysis (Figure 4A) indicated that Group D lighting significantly enriched the expression of genes involved in these pathways [34]. Specifically, 5 DEGs enriched in the phenylpropanoid pathway and 12 DEGs enriched in the flavonoid biosynthesis pathway were identified (Figure 4B). FPKM data showed a progressive increase in both the number and expression levels of upregulated genes in Group D with the extension of refrigeration time, including PAL (*BraA05g03587P*)*,* 4CL *(BraA03g03809P*, *BraA07g03136P*, and *BraA05g02594P*), ANS (*BraA01g01319P*), CHS (*BraA02g00513P*, *BraA03g00571P*, and *BraA10g02342P*), and FLS (*BraA02g04453P*, *BraA06g02703P*, and *BraA10g02681P*) compared with CG0. By days 4 and 6, these genes were significantly upregulated (*p* < 0.05). Conversely, genes such as 4CL (*BraA03g03809P*, *BraA07g03136P*, and *BraA05g02594P*), CYP73A (*BraA03g01531P*), and CYP75B1 (*BraA10g02723P*) were downregulated in the control group (Group A) as refrigeration time increased. These results suggest that Group D lighting stimulates the expression of PAL (*BraA05g03587P*), which consequently activates downstream genes involved in polyphenol biosynthesis [35], including CHS (*BraA02g00513P*, *BraA03g00571P*, and *BraA10g02342P*), CYP75B1 (*BraA10g02723P*), F3H (*BraA09g04185P*), and 4CL *(BraA03g03809P*, *BraA07g03136P*, and *BraA05g02594P*). In summary, under the chilled storage conditions of Group D, it is possible to increase the expression levels of genes related to polyphenol synthesis in pak choi (Figure 4A), thereby increasing the TPC. An increase in TPC can improve a vegetable’s stress resistance and antioxidant capacity.

#### 3.3.4. Key Gene Expression Related to Ascorbic Acid Synthesis

**Figure 5:**
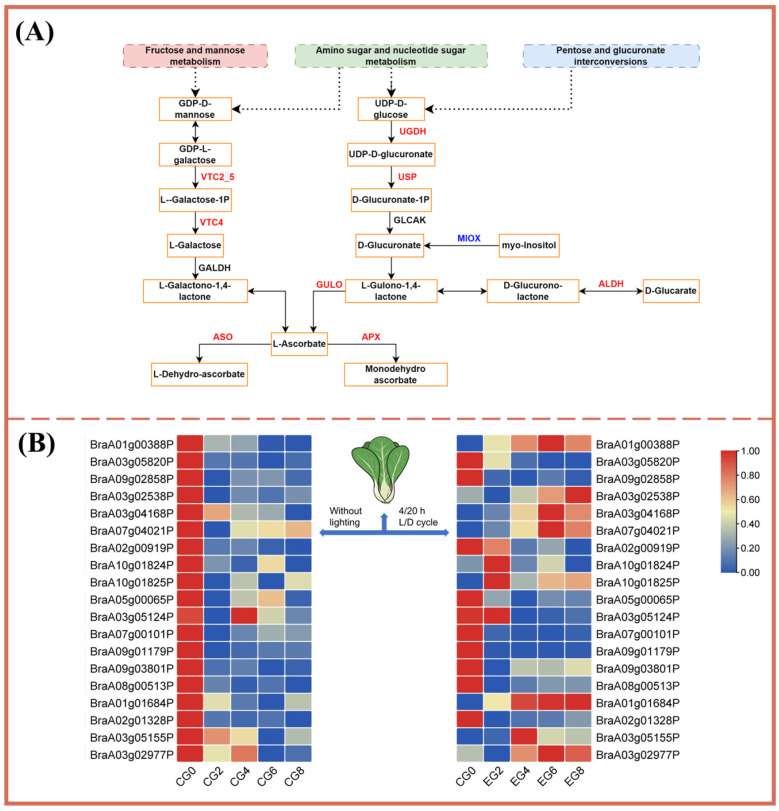
Effects of the lighting regime of Group D on gene expression involved in ascorbate and aldarate metabolism in pak choi. (**A**) Genes associated with ascorbate and aldarate metabolism. (**B**) Heat map of DEG changes in ascorbic acid and aldose metabolism during refrigeration.

Exposure to LED light with a 4/20 h L/D cycle (Group D) resulted in elevated AAC by day 8 relative to baseline. Transcriptomic analysis indicated that this increase was driven by the upregulation of key genes involved in ascorbate and aldarate metabolism [36]. In Group D, genes such as VTC2_5 (*BraA01g01684P*, *BraA02g01328P*, and *BraA03g05155P*), VTC4 (*BraA03g02977P*), and APX (*BraA03g02538P*, *BraA03g04168P*, and *BraA07g04021P*) were upregulated (Figure 5A), whereas these genes exhibited downregulation in the control group (Group A) over the same storage period. Although the expression levels of MIOX (*BraA03g05124P*, *BraA07g00101P*, and *BraA09g01179P*) and ASO (*BraA02g00919P*, *BraA10g01824P*, and *BraA10g01825P*) were downregulated in both groups, their reduction was less pronounced in Group D. These findings demonstrate that the application of the Group D lighting regime induces the expression of metabolic genes associated with ascorbate and aldarate metabolic pathways (Figure 5A), providing a molecular basis for the observed increase in ACC during storage.

#### 3.3.5. Key Gene Expression Related to Chlorophyll Synthesis

**Figure 6:**
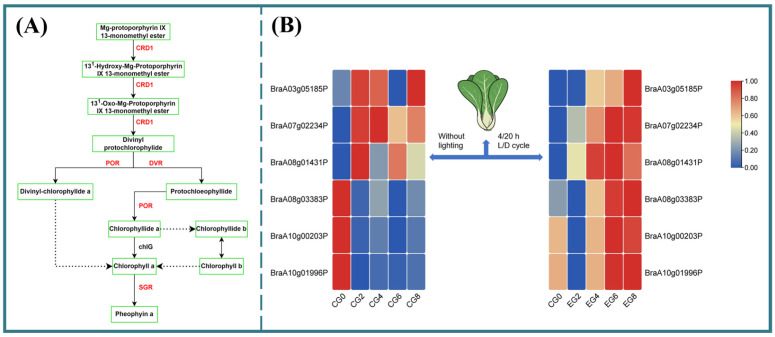
Effects of lighting regime of Group D on gene expression involved in chlorophyll metabolism in pak choi. (**A**) Genes associated with Chlorophyll metabolism in pak choi. (**B**) Heat map of DEG changes in porphyrin metabolism during the refrigeration.

Chlorophyll synthesis is intricately linked to porphyrin metabolism (Figure 6A). Transcriptome analysis (Figure 6B) revealed that key genes in this pathway including CRD1 (*BraA07g02234P*), POR (*BraA03g05185P*, *BraA08g03383P*, and *BraA10g00203P*), and DVR (*BraA10g01996P*) were significantly upregulated in Group D compared to the control during chilled storage [32]. By day 8, expression levels of CRD1 (*BraA07g02234P*), POR (*BraA03g05185P*), and DVR (*BraA10g01996P*) were 3.52-, 3.12-, and 1.30-fold higher, respectively, in Group D (*p* < 0.05), whereas these genes were progressively downregulated in the control group with extended refrigeration (Figure 6A). These findings confirm that the LED regime with a 4/20 h L/D cycle enhances the expression of chlorophyll biosynthetic genes, thereby promoting chlorophyll retention and delaying pak choi leaf yellowing during refrigerated storage. Collectively, these results suggest that Group D lighting can be integrated into postharvest protocols to delay senescence in pak choi by modulating key biosynthetic and metabolic pathways, ultimately enhancing both stress resistance and the preservation of visual and nutritional quality [37]. These insights are particularly valuable for optimizing LED lighting strategies in combination with refrigeration for fruit and vegetable storage in household settings.

In summary, under the lighting regime of Group D, the expression of key genes involved in phenylpropanoid, ascorbate and chlorophyll metabolisms was increased relative to the control group, resulting in greater accumulation of these compounds.

## 4. Conclusions

This study demonstrates that the application of different LED lighting regimes, in combination with refrigeration, significantly affects both the appearance and nutritional quality of pak choi. The 4/20 h L/D cycle was more effective under equivalent DLI conditions, as evidenced by enhanced L* values, increased TCC, and reduced a* values, resulting in a fresher appearance during storage. Furthermore, the 4/20 h light/dark cycle exhibited the advantages of low energy consumption, high feasibility, and low cost, making it suitable for large-scale application in household and commercial refrigerated storage to maintain the quality of pak choi and other leafy vegetables. Transcriptome analysis revealed upregulation of six key genes involved in chlorophyll synthesis—most notably CRD1, POR, and DVR—under this lighting regime. The observed accumulation of total phenolics and ascorbic acid in Group D was linked to increased expression of genes in the phenylpropanoid biosynthesis (PAL, 4CL, CHS, and FLS) and ascorbate and aldarate metabolism (VTC2_5, VTC4, and APX) pathways. These findings elucidate the molecular mechanisms by which the optimal lighting strategy preserves the appearance and nutritional attributes of pak choi, providing a scientific basis for selecting effective LED lighting protocols to improve the quality of fruits and vegetable during preservation, especially in household refrigeration systems and commercial settings.

## Figures and Tables

**Table 1:** Effects of different light mode treatments on the color of pak choi.

Group	Color Parameters	Storage Time (d)				
		0	2	4	6	8
Group A	L*	40.24 ± 0.66 ^a^	39.40 ± 1.24 ^a^	39.64 ± 1.32 ^a^	39.62 ± 1.07 ^a^	39.46 ± 0.48 ^a^
	a*	−9.61 ± 0.32 ^b^	−8.67 ± 0.17 ^a^	−9.07 ± 0.49 ^ab^	−8.72 ± 0.04 ^a^	−8.85 ± 0.51 ^a^
	b*	11.46 ± 0.48 ^a^	11.98 ± 0.93 ^a^	12.65 ± 1.34 ^a^	12.91 ± 1.22 ^a^	13.01 ± 0.36 ^a^
Group B	L*	41.08 ± 0.13 ^a^	41.26 ± 0.50 ^a^	41.38 ± 0.79 ^a^	41.53 ± 0.66 ^a^	40.78 ± 0.31 ^a^
	a*	−8.71 ± 0.05 ^ab^	−8.34 ± 0.28 ^a^	−9.19 ± 0.88 ^ab^	−9.58 ± 0.71 ^b^	−8.70 ± 0.36 ^ab^
	b*	11.47 ± 0.47 ^a^	11.41 ± 0.87 ^a^	11.33 ± 0.73 ^a^	10.79 ± 0.44 ^a^	10.56 ± 0.24 ^a^
Group C	L*	41.36 ± 0.36 ^a^	41.41 ± 0.22 ^a^	41.33 ± 0.35 ^a^	41.24 ± 0.30 ^a^	41.19 ± 0.68 ^a^
	a*	−8.87 ± 0.32 ^a^	−8.82 ± 0.42 ^a^	−9.30 ± 0.38 ^a^	−8.98 ± 0.30 ^a^	−9.11 ± 0.31 ^a^
	b*	11.16 ± 0.41 ^a^	11.28 ± 0.92 ^a^	12.07 ± 0.40 ^a^	11.82 ± 0.19 ^a^	11.66 ± 0.15 ^a^
Group D	L*	41.36 ± 0.35 ^b^	43.20 ± 1.45 ^ab^	42.20 ± 1.60 ^ab^	43.20 ± 2.69 ^ab^	45.16 ± 1.56 ^a^
	a*	−8.71 ± 0.05 ^a^	−10.32 ± 1.33 ^b^	−12.38 ± 0.79 ^c^	−12.73 ± 0.33 ^c^	−13.37 ± 0.80 ^c^
	b*	11.79 ± 0.13 ^a^	11.12 ± 0.78 ^a^	11.07 ± 0.72 ^a^	11.84 ± 0.67 ^a^	10.62 ± 1.02 ^a^

Note: With a row, different superscript letters indicate significantly different (*p *< 0.05).

## Data Availability

The original contributions presented in this study are included in the article. Further inquiries can be directed to the corresponding author.
